# The Hospital Sunday

**Published:** 1901-06-15

**Authors:** Frank Green

**Affiliations:** Lord Mayor


### THE HOSPITAL SUNDAY.

### THE LORD MAYOR'S APPEAL.

The Mansion House, London, E.C.
June 11th, 1901.

To the Editor of *The Hospital.*

SIR,—The approach of "Hospital Sunday" must be my plea for urging, as President and Treasurer of the Fund, that the public should respond, in a liberal spirit, to the appeal which will be made to them on Sunday next in all the churches and chapels of the Metropolis on behalf of the hospitals and dispensaries for the treatment of the sick poor.

This year the council have 201 institutions seeking to participate in the Fund. The needs of all these charities, if only to reimburse them for last year's outlay on the London poor, would not be covered by £100,000.

There is happily no necessity for me to enlarge upon the merits of these institutions, their readiness in relieving those who are afflicted, the benefits they confer on the more fortunate by checking the spread of disease, and their success in mitigating the exceptionally sad consequences of illness when combined with poverty.

But I desire to point out the unique character of this annual collection, and the opportunity which it affords for unostentatious and practical sympathy with a cause which is Christian and self-sacrificing beyond dispute.

Every inhabitant of the Metropolis, irrespective of creed, is offered the privilege of easily gratifying his instinctive sympathy with the suffering poor; he is not subjected to pressure, and of imposture there can be no fear.

Should any of your readers be absent from public worship, or not in London next Sunday, they may remit their contributions to me at the Mansion House.

I am, Sir,
Your obedient servant,
FRANK GREEN, Lord Mayor.

P.S.—I have just received a further generous donation of £10,000 from Mr. George Herring, who in his letter says:—

"I hope your receipts this year will make a record one, as the Hospitals and Convalescent Homes are greatly in need of Funds, and it must be true charity to help our poor through sickness to health."

